# Time-resolved double-slit interference pattern measurement with entangled photons

**DOI:** 10.1038/srep04685

**Published:** 2014-04-28

**Authors:** Piotr Kolenderski, Carmelo Scarcella, Kelsey D. Johnsen, Deny R. Hamel, Catherine Holloway, Lynden K. Shalm, Simone Tisa, Alberto Tosi, Kevin J. Resch, Thomas Jennewein

**Affiliations:** 1 Institute of Physics, Faculty of Physics, Astronomy and Informatics, Nicolaus Copernicus University, Grudziadzka 5, 87-100 Torun, Poland; 2 Dipartimento di Elettronica, Informazione e Bioingegneria, Politecnico di Milano, Piazza Leonardo da Vinci 32, I-20133 Milano, Italy; 3 Institute for Quantum Computing and Department of Physics and Astronomy, University of Waterloo, Waterloo, Ontario, N2L 3G1, Canada; 4 National Institute of Standards and Technology (NIST), 325 Broadway, Boulder, CO 80305, USA; 5 Micro Photon Device S.r.l., Via Stradivari 4, I-39100 Bolzano, Italy

## Abstract

The double-slit experiment strikingly demonstrates the wave-particle duality of quantum objects. In this famous experiment, particles pass one-by-one through a pair of slits and are detected on a distant screen. A distinct wave-like pattern emerges after many discrete particle impacts as if each particle is passing through both slits and interfering with itself. Here we present a temporally- and spatially-resolved measurement of the double-slit interference pattern using single photons. We send single photons through a birefringent double-slit apparatus and use a linear array of single-photon detectors to observe the developing interference pattern. The analysis of the buildup allows us to compare quantum mechanics and the corpuscular model, which aims to explain the mystery of single-particle interference. Finally, we send one photon from an entangled pair through our double-slit setup and show the dependence of the resulting interference pattern on the twin photon's measured state. Our results provide new insight into the dynamics of the buildup process in the double-slit experiment, and can be used as a valuable resource in quantum information applications.

While the double-slit experiment can be used to demonstrate the wave-like nature of quantum particles with mass, it can also be used to show the particle-like nature of light. Double-slit experiments with photons have been carried out using relatively slow exposing charge-coupled device (CCD) cameras^1^,^2^,^3^ and by scanning a single-photon detector through a detection plane^4^, which cannot simultaneously record full spatial and temporal information. In our setup, we use an array of 32 single-photon avalanche diodes (SPAD)^5^,^6^ as a detection “screen” for our double-slit setup. Using this SPAD array in our interference setup, we are able to observe the buildup of the double-slit interference pattern with high resolution in both space and time.

Our experimental setup, shown in Fig. 1, uses photon pairs generated at 842 nm and 776 nm via the nonlinear process of spontaneous parametric downconversion (SPDC)^7^. The 776 nm photon acts as a trigger to herald the presence of the 842 nm photon^8^. The 842 nm photon is coupled into a single-mode fibre, and a polarization controller prepares the state in an equal superposition of horizontal (H) and vertical (V) polarizations. This is then outcoupled, resulting in a free-space Gaussian spatial mode with a waist of 1.3 mm. This beam is collimated and sent to a polarization-based double slit composed of a calcite beam displacer. The birefringence of this crystal results in the displacement of horizontally polarized photons by 3.68 mm with respect to the vertically polarized photons. The beam displacer maps the polarization state of a photon into a spatial state, which is encoded in its path. These two paths are analogous to a double-slit apparatus. They are orthogonally polarized and thus carry distinguishing information, which is erased by a polarizer set at 45 degrees. A compensating crystal (CC) is placed after the beam displacer to make the two path lengths equal, and a series of lenses maps the interference pattern onto the SPAD array.

Each of the 32 detectors in the SPAD array records the arrival time of single photons with a timing uncertainty of about 150 ps, which is the combined timing jitter of the detectors and time tagging logic. Fig. 2(a) shows the arrival times of the first 200 detection events passing through the slits. The accumulation of these events results in an interference pattern, as shown in Fig. 2(b–d). After the detection of 2000 photons, the interference pattern becomes very clear, with a visibility of 93(2)%. This visibility is not perfect as a result of inexact compensation of the two path lengths. A movie and additional measurements using a coherent source can be found in Supplementary Information.

Our ability to accurately measure the arrival times of photons allows us to test the predictions of an alternative corpuscular theory, designed to explain the phenomenon of interference without wave-particle duality^9^. In this theory, detectors are modelled as deterministic learning machines, which are able to reproduce the interference pattern after many photon detections. The detectors' internal states update after each photon detection (see discussion in Supplementary Information), improving their knowledge of the pattern.

Using two statistical methods and the measured buildup of the interference pattern, we examine the predictions of this corpuscular theory and quantum mechanics. The coefficient of determination^10^, *R*^2^, allows us to evaluate how well each model predicts the final interference pattern with increasing detection number, while the likelihood ratio test^11^, Λ, allows us to compare the two models.

We begin by calculating the coefficient of determination, *R*^2^, to see how quickly the measured data, the corpuscular model and quantum mechanics each reproduce the final interference pattern. This pattern is derived from classical wave mechanics, and intensity is used as the only fit parameter (see analysis of interference pattern in Supplementary Information). Our experimental data gives us *R*^2^ = 0.96 after 190(5) detections, as shown in Fig. 3(a). This tells us that the interference pattern is clearly visible after only 190 detection, which we then use as a reference for comparison with the two models. Next, we use the Monte Carlo method to run 10^5^ numerical simulations of 1 … 2000 photon detections for quantum mechanics and the corpuscular model. The statistics of *R*^2^ for these simulations are shown in Fig. 3(a). Although both quantum mechanics and the corpuscular model eventually predict the final pattern very well, they require 200(5) and 1000(10) photons, respectively, to achieve *R*^2^ = 0.96. While it is clear that these statistics for the quantum mechanical simulations and experimental data have similar trends, the coefficient of determination cannot conclusively say which model is better.

In order to compare the two methods, we perform a likelihood ratio test. This test tells us which model is better at reproducing the observed data (see discussion in Supplementary Information). First, we calculate the probability distribution of photon detections based on quantum mechanics. We then numerically simulate the corpuscular model 2.6 × 10^6^ times using the best algorithm^9^ to obtain its detection probability distribution, which is dependent on the number of detected photons. In contrast, the quantum mechanical distribution has no such dependence. Next, we calculate how likely it is that our experimental data emerges from these probability distributions and compare them using the likelihood ratio, Λ. As long as log Λ > 0, we can say that quantum mechanics is better than the corpuscular model. Since log Λ ≥ 0.83 for all points in Fig. 3(b), we conclude that quantum mechanics is a better indicator of the behaviour seen in nature.

In a second experiment, we use our setup with a Sagnac-type source^12^ to generate polarization-entangled photons in the state 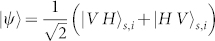 with fidelity 0.94. Here *s*, *i* represent the signal and idler photons. The orthogonal polarization states of the 842 nm signal photon, |*H*〉 and |*V*〉, are transformed into the spatial states |↑〉 and |↓〉 by the calcite crystal. These refer to the two possible paths through the beam displacer. The resulting entangled state is 
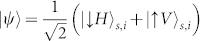
. The 776 nm idler photon is sent to a polarization analyzer, which consists of waveplates, a polarizing beamsplitter and two detectors (see Fig. 1). The orientation of the HWP is set such that detection by D1 and D2 correspond to projection on 
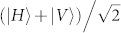
 and 
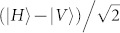
, respectively.

After taking data for 60 s, we filter the detection events by choosing detections at either D1 or D2 as the trigger. If we choose D1 as the trigger, we herald the state 
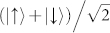
, which leads to the interference fringes shown in Fig. 4(a). Similarily, triggering by detection at D2 heralds 
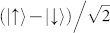
, resulting in a complementary interference pattern. The fringes are complementary because of the phase difference between the states heralded by D1 and D2. If we instead choose to herald using D1 *or* D2 without distinguishing between the two, there is no interference pattern. This is because we effectively ignore the polarization state of the trigger photon, leaving the signal photon in a mixed state. This can be seen as a nonlocal manifestation of a quantum eraser^13^,^14^,^15^.

Because the photons are entangled, the phase of the interference pattern is correlated with the polarization state of the signal photon. To show that we indeed have entanglement between spatial and polarization degrees of freedom, we rotate the QWP in the polarization analyser. The resulting effect on the fringes are shown in Fig. 4 (b,c). The phase of the pattern is clearly dependent on the polarization state of the trigger photon. In contrast, the polarization state of the trigger photon would have no effect on the phase of the interference pattern if these were non-entangled pairs. This heralding can also work in reverse. By post-selecting on a particular point in the interference pattern, it is possible to prepare the idler photon in a specific polarization state. Such a flexible remote state preparation could be very helpful in photonic quantum information processing.

The double-slit experiment, which is at the “heart of quantum mechanics”, has played a central role in our understanding and interpretation of quantum theory^16^. Now, over two hundred years after the first experiments by Thomas Young^17^,^18^, our results provide the most complete picture of single-photon interference to date.

In the future, our measurement techniques will dramatically decrease the difficulty of directly measuring the wave-function of a system by performing weak measurements^19^,^20^. Additionally, these will allow us to herald a variety of polarization states in a multiplexed fashion, as well as facilitate the encoding and transfer of information using the hyper-entanglement of the spatial, temporal and polarization degrees of freedom^3^,^21^,^22^.

## Methods

### Experimental setup

The details of the Sagnac-type source of photon pairs are described in Ref. 12, with a few modifications. The pump is a 404 nm laser diode (Toptica Bluemode), and the down conversion crystal is a 30 mm PPKTP crystal phasematched to produce photons at 776 and 842 nm. The output of C1 has beam waist 1.3 mm. The calcite crystal is 41 mm long, and the compensation crystal is 5 mm long. Lens L1 is plano-convex (f = 150 mm), lens L2 is aspherical (f = 11 mm) and lens L3 is a plano-convex cylindrical (f = 25 mm). D1 and D2 are Perkin Elmer SPCM-AQ4C single photon detectors. The photon source produced around 2 × 10^6^ photon pairs/second which resulted in around 36 × 10^4^ fiber coupled pairs/second. Then the transmission of the calcite system decreased this number to approximately 72 × 10^3^, which results in around 2000 detected coincidences/second. The SPAD array detector dark count rate gives rise to approximately 5 accidental coincidences/second. All 32 channels of the SPAD array are recorded individually as time tags by two logic units (UQDevices).

### SPAD array

The SPAD array is a 32 × 1 array of single-photon avalanche diodes^5^,^6^, with pixel pitch of 100 *μ*m and photon detection efficiency 5% in the range 770–840 nm. It has active area diameter of 50 *μ*m and a dark count rate of 100 counts/s per pixel. For technical reasons, we use 28 of the pixels.

## Author Contributions

P.K., L.K.S. and T.J. led the experimental design. P.K., C.S., K.D.J. and D.R.H. performed experiments. P.K., K.D.J. and C.H. analyzed data. C.S., S.T. and A.T. supplied detection equipment. D.R.H. and K.R. provided entangled photon source. All authors wrote and reviewed the manuscript.

## Supplementary Material

Supplementary Information Supplementary information

Supplementary Information

## Figures and Tables

**Figure 1:**
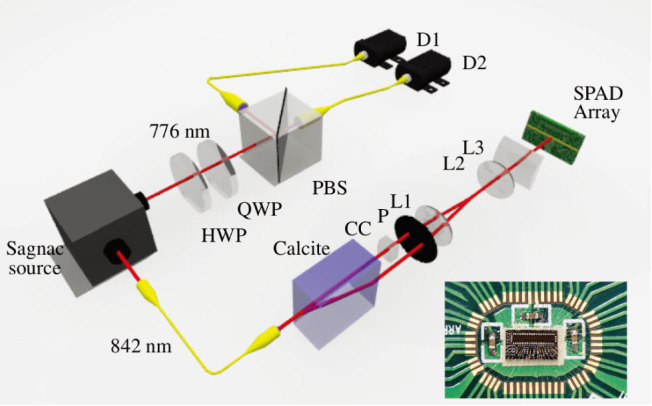
Experimental setup. The Sagnac-type source produces photon pairs. One photon is coupled into single-mode fiber. A birefringent calcite crystal displaces photons with horizontal polarization, and a crystal (CC) compensates for path length difference. A polarizer (P) erases any distinguishing information about the photons. Two lenses (L1 and L2) determine beam size, and a third lens (L3) focuses the beam vertically onto the SPAD detectors. The other photon is sent through a polarization analyser consisting of a half wave plate (HWP), quarter-wave plate (QWP) and polarizing beamsplitter (PBS). It is then coupled into one of two single-mode fibres connected to detectors (D1 and D2). The inset shows a photo of the 32-pixel SPAD array^5^,^6^.

**Figure 2:**
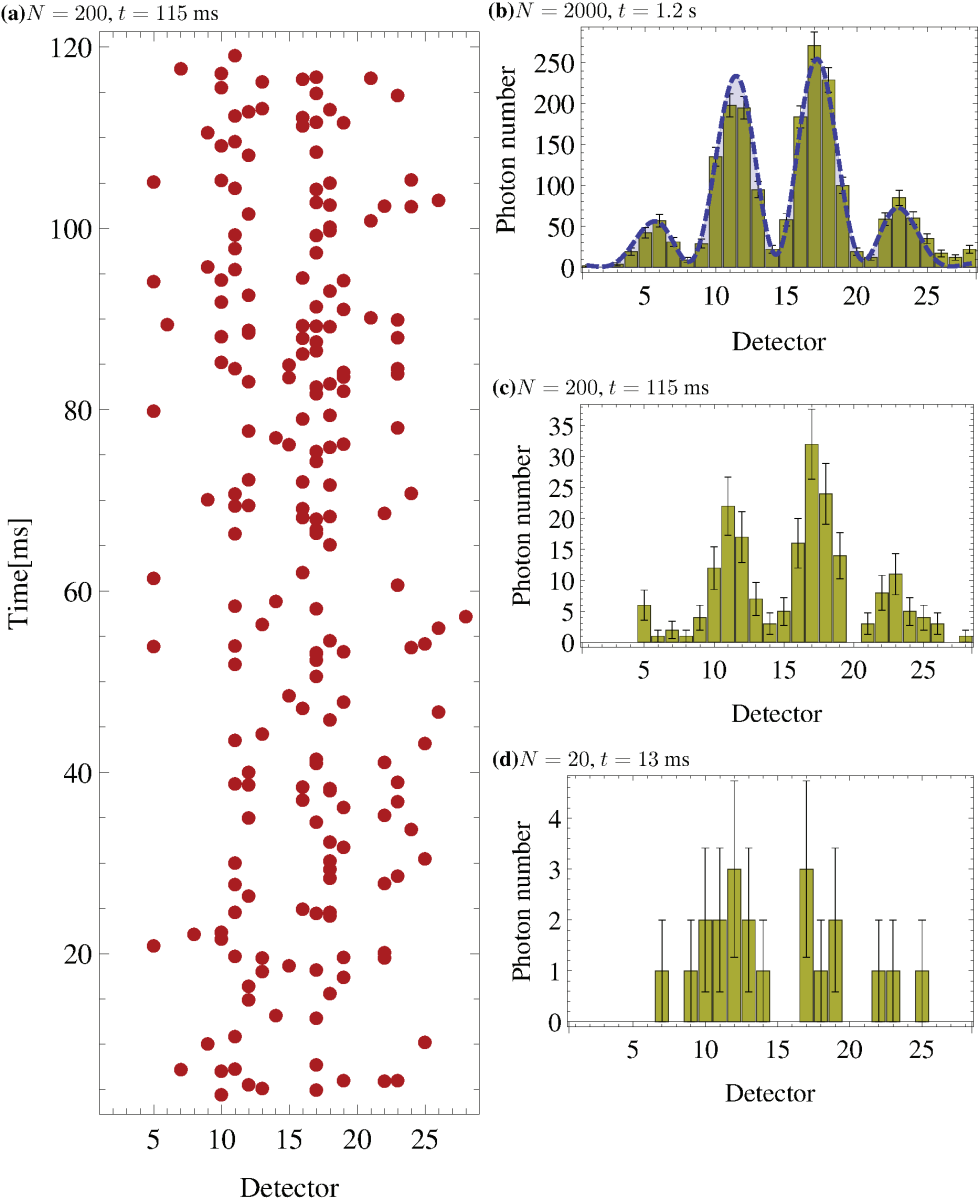
Interference pattern buildup. Panel (a) shows first 200 heralded counts in time, and panels (b–d) depict the statistics of the first 2000, 200 and 20 heralded detections.

**Figure 3:**
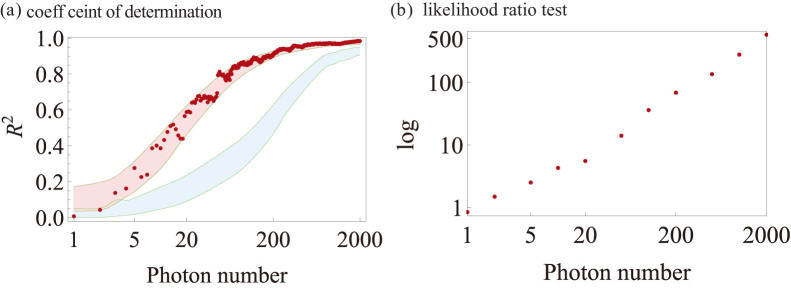
Statistical tests. (a) Coefficient of determination. For a given photon number, the statistics of *R*^2^ is generated after 10^5^ numerical Monte Carlo simulations for the corpuscular and quantum mechanical models. The red (blue) belt shows 50% of the most frequent values of *R*^2^ for the case of the corpuscular (quantum mechanical) model. (b) Likelihood ratio test. The smallest likelihood ratio value is log Λ = 0.83, which shows that quantum mechanics is a better indicator of the behaviour seen in nature.

**Figure 4:**
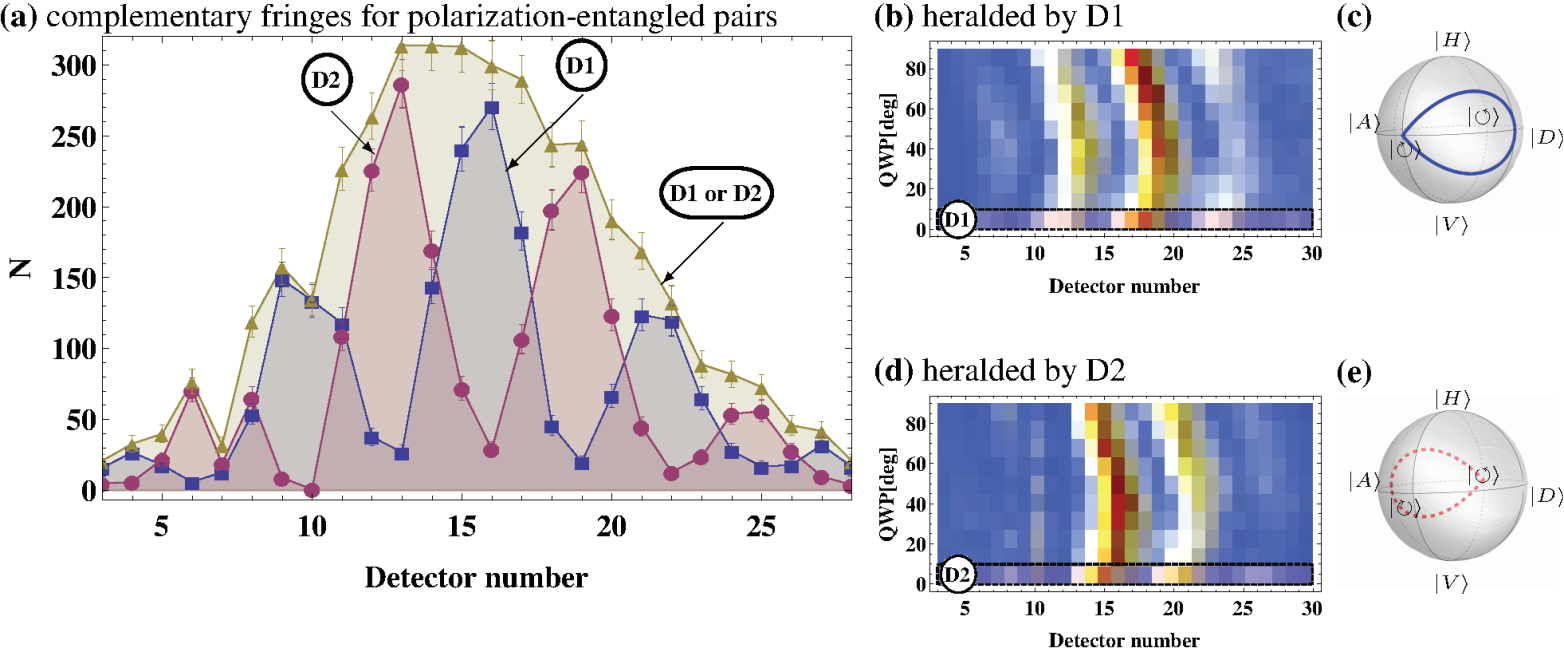
Interference. (a) The round (square) points show the interference pattern of the first 2000 photons heralded by a |*V*〉(|*H*〉) polarized photon. The triangular points show the envelope that results from heralding by either polarization. Limitations of electronics resulted in fewer coincidences at detectors 7 and 10. (b,d) Interference pattern fringes move as the phase is changed remotely by the QWP. The measurements are taken every 10-degree rotation. See Supplementary Table I for the visibilities of each set of measurements. (c,e) The trajectory of the Bloch vector related to the remotely prepared states heralded by (c) D1 and (e) D2.
