# Supplementary material for: Time-resolved double-slit interference pattern measurement with entangled photons
Source: Sci Rep. 2014 Apr 28;4:4685. doi: 10.1038/srep04685 (PMC4001097; doi:10.1038/srep04685)
Supplement: Supplementary Information [file srep04685-s1.pdf]

Time-resolved double-slit interference pattern measurement with entangled photons

Piotr Kolenderski,[1,*] Carmelo Scarcella,[2] Kelsey D. Johnsen,[3] Deny R. Hamel,[3] Catherine Holloway,[3] Lynden K. Shalm,[3,4] Simone Tisa,[5] Alberto Tosi,[2] Kevin J. Resch,[3] and Thomas Jennewein[3]

[1]*Institute of Physics, Faculty of Physics, Astronomy and Informatics, Nicolaus Copernicus University, Grudziadzka 5, 87-100 Torun, Poland*
[2]*Dipartimento di Elettronica, Informazione e Bioingegneria, Politecnico di Milano, Piazza Leonardo da Vinci 32, I-20133 Milano, Italy*
[3]*Institute for Quantum Computing and Department of Physics and Astronomy, University of Waterloo, Waterloo, Ontario, N2L 3G1, Canada*
[4]*National Institute of Standards and Technology (NIST), 325 Broadway, Boulder, CO 80305, USA*
[5]*Micro Photon Device S.r.l., Via Stradivari 4, I-39100 Bolzano, Italy*

Appendix A: Supplementary information

1. Movie

A short movie with a description of the results of this paper can be found accompanying the paper at http://www.nature.com/srep/index.html

2. Additional measurements – Coherent source

Another experimental setup depicted in Fig. S1 has three components: an attenuated laser as a single photon source, a slit system [1] and a SPAD array detector [2, 3].

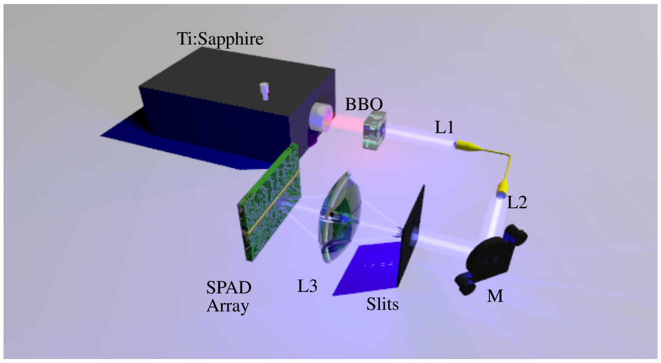

FIG. S1. Attenuated coherent states. The Ti:Sapphire laser pumps the BBO. Photons are coupled into single-mode fiber using lens L1 and collimated by L2 before passing through the double slits. Lens L3 focuses the beam on the SPAD array.

The attenuated coherent states experiment is based on fiber-coupled frequency-doubled 1 ps pulses at 396 nm attenuated such that the SPAD array detects approximately 200 photons/s. This results in 0.06 average photons per pulse which, assuming Poissonian statistics, makes the probability of more than one photon arriving at the slit at the same time negligible.

The Ti:Sapphire laser outputs 792 nm pulses, and is used to pump a 2 mm BBO crystal. Single mode fiber and collimating lens L2 output a gaussian beam with a radius of 0.85 mm (FWHM). Lens L3 has a focal length of 10 cm. The slits are 500μm high, 30μm wide and separated by 100 μm.

Lens L2 is chosen such that the impinging photons' spatial mode size is much larger than the slits' characteristic size. This results in a uniform illumination and transmission of 4%.

After passing through the slits, the photon propagates through lens L3 and is detected by a SPAD array in the focal plane, where the interference pattern is formed. The photons' spatial and temporal modes, in addition to the optics, are chosen such that the interference pattern's characteristic size is comparable to the dimensions of half of the array. This, in conjunction with the high quantum efficiency of the SPAD array at this wavelength, which is 40%, allows an optimal signal-to-noise ratio at the single-photon level. It also allows the expected interference pattern minima (maxima) to coincide with odd (even) numbered SPAD array pixels.

The timing information of a detected photon is recorded only if it is coincidental with a reference pulse from the Ti:Sapphire pulsed laser, thus reducing background noise from dark counts and stray light. The timing resolution of the electronics and SPAD array result in a detection window of 312 ps and an effective 6 total dark counts/s.

We observe the buildup of the interference pattern in time, as seen in Fig. S2. Note that odd-numbered SPAD pixels detect significantly fewer photons than even-numbered pixels, which correspond to the minima and maxima of the interference pattern. The contrast between the neighbouring pixels is clearly visible after only 20 photon detections, indicating the existence of some sort of interference pattern from the beginning of the measurement.

Additional measurements are done for three slits using attenuated coherent states. The imaging optics are adjusted to fit the SPAD array and interference pattern characteristic dimensions. The consecutive detection events as a function of detector number and time, as well as the histograms of the recorded time tags, are depicted in Fig. S3.

Appendix B: The corpuscular model

The corpuscular model of the double-slit experiment [4] gives an alternative description for the buildup of the interference pattern. The detectors are based on deterministic learning machines, whose internal states are updated with each

* kolenderski@fizyka.umk.pl

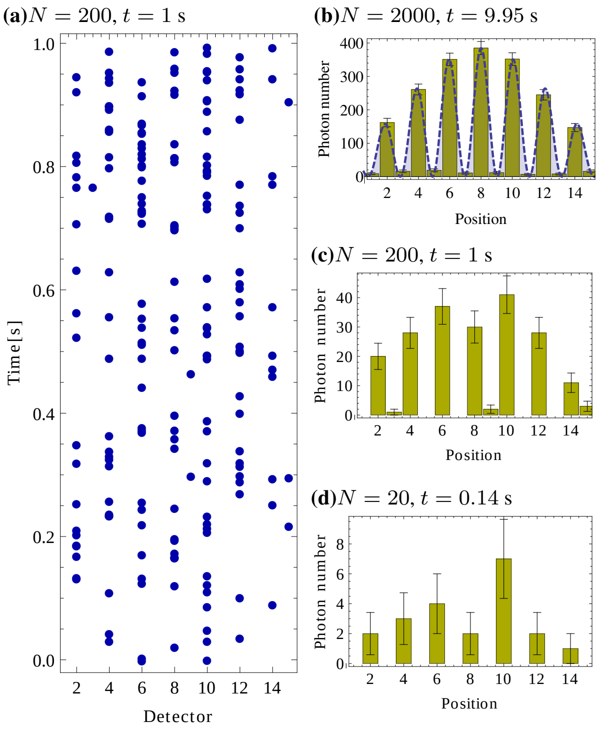

FIG. S2. Interference pattern buildup for 2 slits. (a) First 200 detections in time and (b-d) statistics of first 20, 200 and 2000 detections are presented.

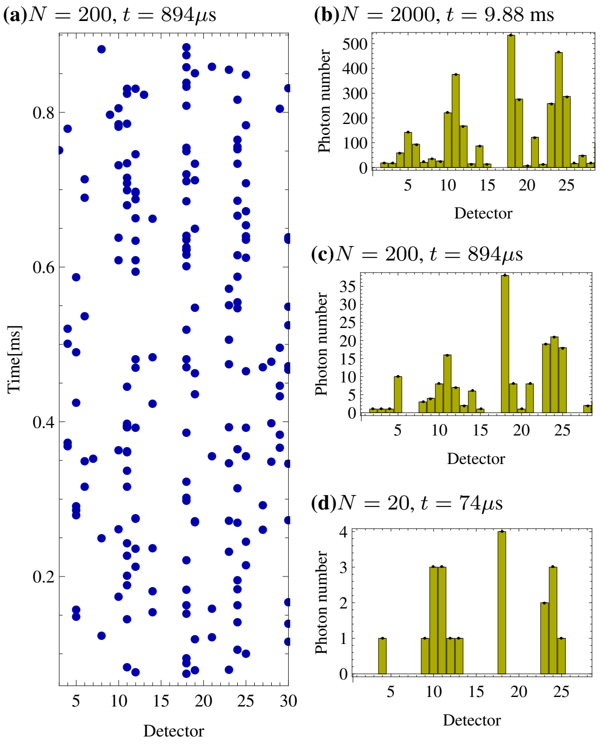

FIG. S3. Interference pattern buildup for 3 slits. (a) First 200 detections in time and (b-d) statistics of first 20, 200 and 2000 detections are presented. Note that due to technical problems, pixels 16 and 17 were disconnected.

”messenger” (photon) detection. The messengers propagate for a specific time after passing through the slits, acquiring a phase ϕ, which then updates the detectors’ states according to the equations [4]:

$$\mu_{k-1} = \gamma(1 - w_{k-1}), \quad \text{(B1)}$$

$$p_k = \mu_{k-1}p_{k-1} + (1 - \mu_{k-1})e_k, \quad \text{(B2)}$$

$$w_k = \kappa w_{k-1} + (1 - \kappa)\frac{||p_k - p_{k-1}||}{2}, \quad \text{(B3)}$$

where κ and γ are constants associated with the detectors, p_k is a parameter that is updated with each detection, and w_k is the internal state of the detector.

In addition to the likelihood ratio test presented in Fig. 3, we comment on few observations on the corpuscular model based on the numerical simulations and our measurements.

We compare this model to the other aspects of our experiment, including complementary fringes and the shifting interference patterns. It is very clearly shown in Fig. 4(a) that the two sets of fringes are extracted from the same measurement data. This situation would confuse the detectors’ learning process, thus telling us that entanglement resides outside of the scope of the corpuscular model.

1. Interference pattern analysis

After passing through the system of a birefringent crystal and polarizer, a wave is in superposition of two spatial gaussian modes displaced by the distance d. Assuming a characteristic radius of w, the corresponding mode functions are:

$$u_{\pm}(x, z = 0) \propto \exp\left(\frac{-(x \pm d/2)^2}{w^2}\right). \quad \text{(B4)}$$

Note that these modes are nearly orthogonal if the displacement d is sufficiently large. In our case, $w = 1.4$ mm and $d = 3.68$ mm and the overlap is $\sqrt{\frac{2}{\pi w^2}}e^{-\frac{d^2}{2w^2}} = 0.0096$.

Next, the standard Fresnel propagation allows to compute the filed in the focal plane of the lens:

$$u_{\pm}(x, z = f) \propto e^{-\frac{\pi x\left(\pi w^2 x \pm 2idf\lambda\right)}{f^2\lambda^2}}. \quad \text{(B5)}$$

By modification of the relative amplitudes and the phases of the two modes, one can prepare any superposition of the following form:

$$\alpha_+ u_+(x, z) + \alpha_- u_-(x, z). \quad \text{(B6)}$$

Equations (2) and (3) allow us to predict the interference patten shape and its dependence on the input state.

Appendix C: Likelihood ratio test

The likelihood ratio test [5] allows to qualitatively evaluate which of the two models is a better predictor of the measured data. This can be done by looking at the ratio, Λ of the probability, $P(D|\mathrm{M1})$, to get a certain set of data, D, under one

model, M1, to the probability, $P(D|\mathrm{M2})$ to get the same data, D, under a second model, M2. It is convenient to take the logarithm of this ratio:

$$\log\Lambda = \log P(D|\mathrm{M1}) - \log P(D|\mathrm{M2}), \tag{C1}$$

where $D = \{k_1, k_2, ..., k_{28}\}$ in our case represents the photon counts distribution measured by the SPAD array (k_i, stands for counts of ith detector) and M $= \{p_1, p_2, ..., p_{28}\}$ is the probability distribution given by a model (p_i is the probability of detecting a photon by ith detector). If $\log\Lambda > 0$, we can say that M1 describes experimental data better than M2 does.

The probability to get a distribution, D, assuming probability distribution, M, is calculated using a multinomial expansion:

$$P(D|\mathrm{M}) = \sum_{k_1+k_2+...+k_{28}} \frac{n!}{k_1!k_2!...k_{28}!} \prod_{1\leq n\leq 28} p_n^{k_n}. \tag{C2}$$

The probability distribution for the quantum mechanics, M1, is computed in the following way. We fit the photon statistics acquired after detection of 98000 photons to the quantum mechanical model. The fitted parameters are intensity, transverse shift and magnification. The remaining setup parameters were fixed to the measured values. The coefficient R^2 is 0.99. Note that within this model the probability distribution does not depend on detected photon number.

This is the feature that differs quantum mechanics and the corpuscular model. To calculate the photon detection probability distribution, M2, for the corpuscular theory, 2.6×10^6 simulations were made for photon number in the range $1 \ldots 2000$. In contrast to quantum mechanics, the probability distribution for this model depends on the number of photons that have been detected.

Appendix D: Interference pattern visibilities

QWP [deg]	Vis., [%], D1	Vis.,[%], D2
0	93±2	96±2
10	94±2	98±2
20	93±2	94±2
30	97±2	92±2
40	94±2	94±2
50	96±2	93±2
60	90±2	87±2
70	84±2	85±2
80	75±2	89±2
90	91±2	94±2
100	93±2	95±2

TABLE I. Visibilities for patterns obtained by QWP rotations, see Fig. 4.

[1] Kolenderski, P. *et al.* Playing the aharon-vaidman quantum game with a young type photonic qutrit. *Phys. Rev. A* **86**, 012321 (2012). arXiv:1107.5828.

[2] Zappa, F., Tisa, S., Tosi, A. & Cova, S. Principles and features of single-photon avalanche diode arrays. *Sensors and Actuators A: Physical* **140**, 103 – 112 (2007).

[3] Scarcella, C., Tosi, A., Villa, F., Tisa, S. & Zappa, F. Low-noise low-jitter 32-pixels cmos single-photon avalanche diodes array for single-photon counting from 300 nm to 900 nm. *Rev. Sci. Instrum.* **84**, 123112 (2013).

[4] Jin, F., Yuan, S., Raedt, H. D., Michielsen, K. & Miyashita, S. Corpuscular model of two-beam interference and double-slit experiments with single photons. *J. Phys. Soc. Jpn.* **79**, 074401 (2010).

[5] Casella, G. & Berger, R. L. *Statistical Inference* (Cengage Learning, 2001), 2 edn.
